# The Nursing Supplement

**Published:** 1889-12-14

**Authors:** 


The Hospital,
December 14, 1889.

Extra Supplemen

### The Nursing Mirror.

All Contributions for this Supplement should be addressed to tne Editor, The Hospital, 139-140, Salisbury Court, Fleet Street, London, E.C., and should have the word "Nursing" plainly written in left-hand top corner of the envelope.

### En Passant.

CHURCH ARMY NURSES.—The Salvation Army is sending out another Indian contingent, but the Church Army has not yet got its special nurses trained and sent to the lepers. It is a pity the Church Army does not train its nurses since it calls them "trained." They have six weeks at Kensington Infirmary, and then live for six weeks at the home in Edgware-road, and wear a shield in the front of their black bonnets. Then they are sent forth as trained district nurses, and wear a small sword in their bonnets as a badge. They do good work, but the Army has no business to call them trained nurses.

THE GRATITUDE OF A DANE.—Not long ago, a Dane was a patient at the West Norfolk and Lynn Hospital, and after he returned cured to Ronne he wrote a letter to the matron, of which the following is a translation: "Dear Miss Brightman,—I thank you very much for the long time I was with you in Lynn, and you must thank all the doctors for their pains—Drs. Dale, Sumpter, and Allinson, who all helped to cure me. Please also thank my faithful nurse Broughten for all her kindness. I now wish that you all may live well and pleasant until I see you again.—Your good friend, Anders Julius Sorth."

A NURSES' CONVERSAZIONE.—This year's British Nurses' Association *soirée*, at the Prince's Hall, was not quite so good as last year's at the Grosvenor, still it was a most enjoyable evening. The guests were received by Miss Thorold, which caused some surprise, especially as Miss Wood was present. Rumour whispered, however, that Miss Wood had resigned her official connection with the British Nurses' Association, hence the manner in which she kept in the background all the evening. The rooms looked bright and cheerful, full of the neat, becoming uniforms we all know so well. Bartholomew's, Great Ormond-street, and the Throat Hospital nurses seemed most in force, though St. John's House sent a fair contingent. The provinces were poorly represented compared with last year; the matron of Cardiff and the matron of Newport we saw, but we missed the army and navy sisters with their striking uniforms. There was not a single red cross on show, though one matron had decorated herself with a huge silver cross and a large Primrose League badge in her efforts to obtain effect. Certainly the nurses looked nicer than the visitors, and we were more than ever convinced that the nursing uniform is the most becoming dress possible. The concert in the Prince's Hall was good; Mrs. Stanley Stubbs played pranks with her remarkably flexible voice, and Miss Nesbit recited with power her "Ballad of Splendid Silence." But the honours of the evening were reserved for Corney Grain, who was greeted with such continuous laughter and applause that it was difficult to hear all he said. During the concert, Mrs. B. Fenwick, Miss Mollett, and one or two others sat in a gallery apart. General regret was expressed at Miss Mollett's approaching departure, all wishing she could have secured a post in England and remained amongst her friends. Mrs. B. Fenwick was wearing Bartholomew's uniform, and looked very well. There were one or two matrons and nurses there who were not in uniform, apparently not desiring to be recognised in their official capacity. The whole of the arrangements for the *conversazione* were excellent, and speak well for the organising power of the secretary, Miss Wood; still, after all its great promises of great deeds, it is astonishing that so far the association has done little but give its members two pleasant evenings and a day at Cambridge.

ARMY NURSES.—The Secretary for War issued a Royal warrant on December 4, which directs that in future candidates for the appointment of acting-superintendent or nursing-sister shall not be accepted unless they are over twenty-five and under thirty-five years of age, and have had at least three years' preliminary training and service combined in a civil hospital. No acting-superintenden or nursing-sister is to continue in the service after the age of sixty.

NORTHAMPTON INSTITUTE.—Last month, Miss Wood delivered an excellent lecture on "Modern Nursing" at the Northampton Nursing Institute, and the Bishop of Leicester, who presided, told the following true tale anent the prejudice often shown against trained nurses: "An eminent divine, and a confirmed bachelor, was taken seriously ill, and his friend called in a trained nurse. 'What is that?' asked the good man, pointing to the lady in her neat costume. The explanation was not sufficient, and the friend had to comply with the invalid's request to 'show her out,' and to send to the barracks for an orderly. An orderly came, and in the arms of the soldier the good man subsequently died."

EPSOM DISTRICT NURSE.—At a meeting held at Epsom Public Hall on December 5 of the Nursing Association, the hon. secretary and treasurer, Mrs. Robertson Rodger, read the report, which stated that the committee presented to the subscribers of the association, with great satisfaction, their second annual report, giving an account of the past year's work among the sick poor of Epsom. During the year 112 patients had been nursed, in addition to which 2,529 visits had been made by the district nurse. The balance-sheet showed that the subscriptions amounted to £125 10s. 6d., and the expenditure £111 14s. 2d. The report was adopted, and Dr. Alexander and others spoke in favour of the work done.

PAUPER NURSES NO MORE.—That will be the cry everywhere soon; a small forward movement is being made at Richmond, but, as usual, those in power now, dislike alterations and bring forward objections. The master (what does he know of nursing?) says he will be satisfied with the trained night nurse, as there is not proper accommodation for more. The committee recommended the appointment of a trained night nurse, at a wage of £25, rising to £30, and an assistant nurse at a wage of £18, rising to £22, to take charge of the children. They also suggested several minor alterations in detail on the premises; advised that when extra nursing assistance was needed, the master should be empowered to obtain the same from a nursing institute; and recommended the establishment of communication by telephone between the wards.

DEATH ON DUTY.—A melancholy event is reported to have occurred in New York. Miss Catherine Coster, twenty-six years of age, head nurse in Dr. Tod Helmuth's private hospital at 41, East Twelfth-street, has died from burns caused by an explosion of ether. Miss Coster went into the hall to pour the contents of one bottle into the other, when one of the bottles exploded and her clothing was set on fire. It is supposed she stood near a lighted gas-jet. The death is also announced of Nurse Peat, late of Grafton-street Fever Hospital, Liverpool, who contracted fever while on duty and died at her post. Two other nurses—Nurse Webb and Nurse Moore—of this hospital are also ill, and not long ago we chronicled the death of another of these nurses. This looks as though the allegations of overwork with regard to Grafton-street Hospital are true.

### Among the Nurse-Dolls at Charing Cross.

*(By an Outsider.)*

THE board-room at Charing Cross hardly knew itself last Saturday. The walls were hidden by draperies of Oriental design, whose rich tones of venetian red and tawny yellow brightened even the greyness of the dull, wet December day. Ferns, palms, and flowers were grouped in the corners, and on the tables were arranged "our dolls." I noticed that every nurse who came to see the show looked upon the little figures as "our dolls," and combined a special affection for the uniform she wore, or had once worn, with a kindly interest in every other. Of course, all uniforms are not equally becoming, but looking at the dresses that are worn now, and comparing with a curious specimen which was shown of the mode of twenty years ago, it must be admitted that dress is one of the things in which the nursing profession has improved, and improved all along the line, in suitability as well as appearance. The judges had not, however, been led away in their decisions by mere prettiness of style and material. The prizes went to the dolls in which neatness of workmanship was combined with the most accurate reproduction of a nurse's dress, with no fanciful alterations for the sake of effect.

There was no doubt of the interest excited by the show. I was amused to see so many members of that mystical body, the Press, and to notice the satisfaction which they, as, a rule, the most *blasé* and cynical of mortals, seemed to find in the puppets. It was quite worth while going to see the representative of the *Daily Telegraph* lending six feet of muscular manhood to the study of Charing Cross dolls, which, charming as they were, were disqualified from the competition, from the fact that Miss Gordon, the matron, was one of the judges. Meanwhile, on the other side of the room, the lady who came from the *Pall Mall Gazette* was making little sketches of the various styles in vogue at some of the principal hospitals. The *Graphic* had, however, been before her in securing drawings of the uniforms in the metropolitan hospitals. Several provincial newspapers were represented, and I saw the *Manchester Examiner*, the *Sheffield Telegraph*, and the *Birmingham Post*, all looking carefully for local dolls.

Meanwhile, what about the dolls themselves. There they were, come from east and west, north and south—from John o' Groats to Lands End, or at least from Aberdeen to Winchester. Scotland was, however, rather poorly represented, and only the County Cork Hospital represented "Poor Ould Oireland." Perhaps, however, our Irish friends will one day determine to have a national exhibition of nursing uniforms all to themselves.

It is greatly to be regretted that prizes could not be given to all the dolls sent in, but since this could not be the following were, after much consideration, adjudged as the most worthy:

SETS OF THREE OR MORE DOLLS: First Prize, 40s., Salop Infirmary, Nurse Jesse. Second Prize, 30s., Radcliffe Infirmary, Nurse Barnes. Third Prize, 20s., Charing Cross in 1879, Nurse Carling. Fourth Prize, 10s., Norfolk and Norwich Nurses' Home, Nurse Taylor.

SETS OF TWO DOLLS: First Prize, 30s., Putney Hospital for Incurables, Nurse Slaughter. Second Prize, 20s., Bradford Infirmary, Nurse Phillips. Third Prize, 10s., Sheffield Infirmary, Miss Rickards. Fourth Prize, 5s., Pendlebury Hospital, Miss Plowon.

SINGLE DOLLS: First Prize, 20s., Swindon Victoria Hospital, Mrs. K. Smith. Second Prize, 10s., National and Metropolitan Association, Nurse Prikler. Third Prize, 5s., Cardiff Infirmary, Miss Davies.

Highly commended were the dolls from the Darlington Hospital, Devon and Exeter Hospital, Birmingham Nurses' Institution.

Some of the competitors had done more than was actually required of them. There was a ward scene sent in from University College, which showed not only the dresses of sister, nurse, and probationer, but two convalescent children whom they had tended. A nurse at Miss Pollock's institution in Weymouth-street sent in a patient she had dressed and bandaged. He was in a very bad case, this patient. His every limb was bandaged with most exquisite neatness; his left arm was in a splint, he wore a patch over one eye, and his head was bandaged as for a broken jaw. In fact, only the toughness and elasticity of his indiarubber constitution gave any hope of his ultimate recovery. It is, of course, impossible to detail all the costumes worn; a mere catalogue would hardly be interesting, and it is possible that readers of THE HOSPITAL in various parts of the country may be able to see the collection for themselves. An urgent request has come from Liverpool that they may be shown there; after that they may go to Glasgow, and probably to other towns as well. Finally, it is their destiny to be kept in a museum of nursing costume in connection with THE HOSPITAL office, where, at some future time, it is to be hoped that many nurses may see them.

To our friends at Charing Cross who gave standing room and a hearty welcome to our dollies; to Mr. E. P. and Miss Warren, who kindly decorated the room; to Mr. J. Aldam Heaton, who lent the pretty Palampore draperies; and to Mr. Buck, of Covent Garden, who lent some lovely flowers, our best thanks are due. And last, but not least, our warmest thanks to our nurse friends all over the country who dressed the dolls for the honour and glory of their respective institutions. Though they cannot all have prizes, though many of them will never know how nearly they did get prizes, they can all have the cheering thought that their labour was widely appreciated, and will be an interest and pleasure to many, we hope, for many a year to come.

### Examination Questions.

THE prize for November has been awarded to Miss Payne, of Bolingbroke House, whose answer is now printed. Nearly fifty answers were received, and the judging was extremely difficult. "Osteon" ran Miss Payne very close, but had not mentioned the difference in the compact and cancellous appearance of bone. Two answers were received from Taunton so exactly alike, it was impossible not to draw the inference that they both got their inspiration from some source such as a lecture or book kept in the hospital. Some learned treatises had to be put aside on account of their length, some because every other word was underlined and there were no stops, some because they could only name three classes of bone, and, lastly, some which arrived after the appointed date. One paper from Carmarthen was beautifully illustrated, but, unluckily, we have no artist on our staff, so preferred verbal descriptions of the different bones. Considerable delay was caused by many of the answers being wrongly addressed, so please note that in future they must be sent to the Editor of THE HOSPITAL, 139-140, Salisbury-court, Fleet-street, London, E.C., the word "Nursing" being written in the left-hand top corner of the envelope. As we are too late to give a question for this month the answers to the following question for January need not reach our office till January 11, 1890: *Question.*—"What should be specially observed in nursing a case of (1) amputation of a limb, (2) operation for cataract, (3) severe burn."

*Prize Answer.*—Describe the composition of bone; name the four classes of bone; and give an example of each.—Bone is composed of about one-third of animal substance, and two-thirds of earthy and alkaline salts. It is one of the hardest structures of the animal body, but possesses a certain degree of toughness and elasticity. When fresh it is of a pinkish-white colour externally, and deep-red within. The exterior of bone is dense and compact, like ivory, and the interior is spongy, and from its resemblance to lattice-work is called cancellous (from the Latin *cancelli*, lattice-work). The dense and the spongy tissues are the same in structure, but differ in the degree of closeness with which their fibres are packed. A tough, fibrous membrane, called the periosteum, covers the exterior of all bones, except the parts covered with articular cartilage. The periosteum forms the means of attachment for the muscles and tendons, but its chief use is to support the bloodvessels, which supply the bone with nourishment. The hollow shafts of the long bones are filled with marrow, which differs in composition at different periods of life, and in different bones. In the shafts of adult long bones the marrow is of a yellow colour, and consists of fine connective tissue supporting fat vesicles, whilst in the articular ends of the long and short bones, and especially in the base of the cranium, it is reddish in colour, and contains very little fat. The medullary membrane, or endosteum, is a thin, delicate, areolar membrane; it lines the hollow shafts of the long bones, and is supplied with numerous small bloodvessels, which reach it through openings numerous and small, at the extremities of long bones, and by one large artery (or sometimes more) which enters a channel about the centre of the shaft called the nutrient foramen. This nutrient artery, usually accompanied by one or two veins, sends branches upwards and downwards to supply the membrane. But there is a certain portion of dense tissue in most bones which is too far removed from the periosteum or the medullary membranes to draw support from them. This part is supplied with bloodvessels, entering channels called the Haversian canals, but besides these there are extremely minute channels called canaliculi, which permeate the whole of the dense tissue; these radiate from the Haversian canals, and are interrupted in their passage to the more distant concentric lamæla by pouring their contents into small oval cavities, called the lacunæ of bone. The Haversian canals are only found in bones of some thickness, but the lacunæ and their corresponding canaliculi are to be found in all parts of a bone however thin. Nerves are distributed freely to the periosteum, and enter the interior of the bone by the nutrient arteries. The four classes of bone—long, short, flat, and irregular; the humerus is an example of the long, the carpus of the short, the scapula of the flat, and the vertebræ of the irregular.

### The National Pension Fund for Nurses.

(From the *Insurance Critic.*)

In another column will be found the annual report, together with the accounts and balance-sheet, presented by the council at the general meeting of the National Pension Fund for Nurses on Tuesday last. This interesting document is well worth perusal.

The National Pension Fund for Nurses is undoubtedly one of the wisest schemes of benevolence than can well be imagined. It is entirely superfluous to say that the trained nurses of the present generation constitute one of the most meritorious and useful classes of modern women. It is impossible to think too well of them, and equally impossible to do too much for them. The public who employ nurses no doubt think that their rate of payment is considerable. Compared with the amounts which used to be paid to the Gamps and Harrises of a former generation, it is considerable; but, compared with the value of the services rendered, it is exceedingly small. That, however, is not the point to which special attention ought to be directed here. The wages paid to trained nurses are totally inadequate as remuneration for the class of women employed. They are not more than sufficient to barely maintain them during their period of active service. That active service is necessarily very short, for nurses, it is found by experience, are superannuated when they are barely past middle age. Hence arises the necessity for some kind of public provision to be made for this exceptionally deserving class.

Actuarially, the inception of a pension fund was a new departure, and a new departure of a peculiarly uncertain kind. For although deferred annuities are amongst the common operations of assurance societies, yet a society devoting itself to the sick maintenance and pensioning of a separate class of individuals and to no other business, is an experience totally new. Under the circumstances, the starting of a pension fund, the bringing in of the beneficiaries, the satisfying of the scientific conditions of the case, and all the other accompaniments of such a departure, was a work demanding peculiar qualifications and energy of the most determined character. It is easy to conceive that, notwithstanding the undoubted value and necessity of such a fund, the failure of the attempt to establish it might have been disastrous and complete. But, thanks to the energy, perseverance, skill, and enthusiasm of the several persons connected with it, the National Pension Fund for Nurses is at this moment one of the most successful insurance societies in this much-insuranced country.

The new association does its work without trenching on ground occupied by any other assurance society; and all the older societies, we are sure, regard it with feelings of kindly approval. It is much to be desired that the appeal, which, we understand, will shortly be made to the public on behalf of the bonus fund, will be justly appreciated and responded to with generous liberality. We repeat that no object which has ever been started for the benefit of an invaluable class better deserves the generous support of the public.

For us to comment on the scientific aspect of the accounts presented is superfluous. A society which has conducted its first year's operations to an issue so successful in every department, for a sum not exceeding its loading and probable profit interest, is phenomenal, and probably without parallel.

### Everybody's Opinion.

[*Correspondence on all subjects is invited, but we cannot in any way be responsible for the opinions expressed by our correspondents. No communications can be entertained if the name and address of the correspondent is not given, or unless one side of the paper only be written on.*]

### OVERWORKED NURSES!

A. E. S. writes: May I again urge the necessity for shorter hours amongst us? It is indeed time that fifteen and sixteen hours in sick wards should be a thing of the past. So much is written just now about the long hours of the "tram drivers and 'bus conductors," will no one say a word for us? Few, we will hope, outsiders know the length of our hours, or surely they would help us. We cannot strike (though one of my own new patients said the other day he thought for himself ten hours' work was too much), for that means loss of work and want of bread; were we *old* nurses to strike there are hundreds of probationers who would be ready, spite the sixteen hours, to step into our places for the sake of the extra wages, and we should be left in the cold, for who cares for a worn-out nurse? [The Pension Fund, most certainly.—Ed.]. We spend our young, strong days working for others; when ill are kindly nursed by our fellow-workers, but if unable to return to work we may do as we can. Surely, from 6.30 a.m.—which means rising at 6 o'clock—until 9 p.m., is too long to be on our legs, and there is precious little time for rest, as patients always want something doing for them. Half an hour for meals is a good allowance, I grant, when we can get it, but fresh air twice a week is far too little for health, and so short-handed are most hospitals, particularly provincial ones, where there are only two nurses—rather nurse and probationer in each ward—that not often can more be had. I think we might receive more consideration at the hands of "our masters," and our lives would be brighter; for why should they be made so dull and hard? why should we work such long hours for so little money? I defy the best paid nurse in England to save £200 in long years of nursing; and who could live on the interest of that sum? How often have I heard a house surgeon exclaim, "I've had such a hard day, I am so tired," when I knew he did not breakfast before 9 o'clock, did perhaps two or three hours' work afterwards, then, after lunch, smoke, sleep, play cards, see a few patients, run round the wards, spend a jolly evening with his friends, and growl at "those blessed patients or nurses," if they called him out. At the same time, we have never sat down from 6.30 to 9 p.m., except for meals—and we are only women! [We shall be glad to hear from other nurses if the hours are equally long anywhere else, or in every case where they exceed ten hours a day throughout the week. "A. E. S." should mention the name of the institution to which she refers.—Ed.]

### AN A B C.—"MY IDEAL NURSE."

A Hospital Nurse writes: The outside world's ideal of a nurse, as given in last week's Hospital, "sweet, sad, sentimental," set me thinking of the qualifications of my ideal nurse. These are some of them. I don't pretend that the list is complete. A nurse should be—

Active, attentive, alert,
Brave, bright, business-like,
Calm, cheery, comfortable,
Docile, diligent, decided,
Energetic, enthusiastic, even-tempered,
Faithful, fearless, far-seeing,
Gentle, good-humoured, generous,
Healthy, hopeful, humble,
Improving, intelligent, indefatigable,
Joyous, judicious, just,
Kind,
Loyal, liberal-minded, light-footed,
Merry, managing, modest,
Neat, notable, nice,
Obedient, orderly, an optimist,
Prompt, patient, painstaking,
Quick-sighted, quiet,
Ready, reliable, resourceful,
Sympathetic, systematic, self-forgetting,
Truthful, thorough, temperate,
Unprejudiced, unassuming, uncomplaining,
Vigilant, vigorous,
Wary, warm-hearted, well-informed,
Excelsior,
Young in heart with a Y Z.

### Notes and Queries.

### Queries.

(30) *Male Nurses.*—Where can they get training? *Nurse L.*

(31) *Home Wanted.*—Where a male surgical patient could be received, Bournemouth preferred. What terms? *Matron.*

(32) *Book on Private Nursing.*—Which is the best book on private nursing. *K.*

(33) *Home for Imbecile.*—Where can an imbecile child, aged eight, be received? The parents are poor and could only pay a small sum. *Constant Reader.*

### Answers.

(30) *Male Nurses.*—The Hamilton Association, 57, Park-street, Grosvenor-square, or Mr. Wilson's Institute, 96, Wimpole-street.

(32) *Book on Private Nursing.*—"The Duties and Conduct of Nurses in Private Nursing," by Dr. Richardson. Published by Field and Tuer, price 1s.

*Massage.*—Can be learned on the Playfair system at the National Hospital, Queen-square, for moderate terms. I know of no place at Bristol. *No-No.*

*Dispensing.*—At the Zenana Medical College, St. George's-road, or at the New Hospital for Women, Marylebone-road, but I don't know exactly what fees. *Jane.*

*Charity.*—Apply to Lady Samuel, 15, Courtfield-gardens, Earl's Court.

### Answers to Correspondents.

"*A Patient of Hospital Nurses*" is requested to send name and address to the Editor. No letters can be published unless this information is forthcoming in the first place as a guarantee of good faith.

### THE SMALLEST THINGS.

The smallest crust may save a human life.
The smallest act may lead to human strife.
The smallest touch may cause the body pain.
The smallest spark may fire a field of grain.
The smallest deed may tell the truly brave.
The smallest skill may serve a life to save.
The smallest drop the thirsty may relieve.
The slightest look may make the heart to grieve.
Naught is so small that it may not contain
The rose of pleasure or the thorn of pain.

### Presentation.

Mr. W. H. Tomlinson, for two and a half years house surgeon at the Oldham Infirmary, lately resigned his appointment to enter upon a private practice at Leek, Staffordshire. On leaving Oldham, Mr. Tomlinson was presented by the Matron (Miss Thompson) and the nurses of the institution with a timepiece, bearing a suitable inscription; by the servants with a dressing case, and by the committee with an engrossed testimonial. After the presentation, the nurses spent a social evening, and gave a small dance.

### Vacancies.

The following vacancies are announced:

### Matrons.

Bath Nurses' Home, £50.
Clifton College Sanatorium.
Dunster and Minehead Hospital.
Kent County Asylum(assistant), £85.

### Nurses.

Aylesbury Infirmary, £25.
Bedfordshire Institute, £26.
Birmingham Orthopœdic Hospital £20.
Blackheath Nursing Institute.
Bridgwater Infirmary, £25.
Central London Throat Hospital, £20.
Chester Deaconess Institute, £24.
City of London Chest Hospital, E., £20.
Dover Hospital.
Eastbourne Institution.
Glasgow Barony Parochial Hospital, £24.
G. W. R. Medical Fund Accident Hospital, £35.
Hackney Infirmary (two), £20.
Jersey Nurses' Institute, £25.
King's Cross Hospital, Dundee, £24.
Leicester Institution, £25.
Liverpool Foundling Hospital.
Loughborough Infirmary.
Macclesfield Infirmary, £24.
Middlesbrough Fever Hospital, £30.
Newcastle City Hospital, £25.
Newport Nursing Institute (several)
North-Western Fever Hospital, £30.
Nottingham Nurses' Institute, £25.
Nursing Institution, 96, 97, 98, Wimpole-street, London, W., Mr. Wilson has several vacancies for thoroughly-trained nurses.
Portsmouth Infectious Diseases Hospital (two).
Royal Albert Hospital, Devonport.
Sheffield Nurses' Home.
Southport Infirmary (night), £20.
Southport Nurses' Home (several).
St. George's Infirmary, W. (three).
St. Helena Home, N.W.
St. John's Institute, Southport, £25.
St. Saviour's Infirmary, S.E. (several), £20.
Swansea Nursing Institute, £25.
Truro Infirmary, £20.
Ventnor Consumptive Hospital, £20.
Western Fever Hospital, £22.
Workhouse Infirmary Nursing Association.

### District Nurses.

Cowbridge House, Malmesbury.
St. Jude's, Herne Hill, £40.
8, St. Margaret's-street, Canterbury.

### Probationers.

Canterbury Nurses' Institute.
Kensington District Nursing Association.
Kent and Canterbury Hospital.
North-West London Hospital.
St. Helens Hospital, Lancs.

### Amusements and Relaxation.

### Third Quarterly Word Competition Commenced October 5th, 1889, ends December 28th, 1889.

**Three Prizes of 15s., 10s., 5s., will be given for the largest number of words derived from the words set for dissection.**

**Proper names, abbreviations, foreign words, words of less than four letters, and repetitions are barred; plurals, and past and present participles of verbs, are allowed. Nuttall's *Standard* dictionary only to be used.**

**N.B.—Word dissections must be sent in WEEKLY, arranged alphabetically, with correct total affixed.**

**The word for dissection for this, the *Eleventh* week of the quarter, being "GUINEA FOWL."**

### Results of Ninth Week.

| Names. | Dec. 5th. | Totals. | Names. | Dec. 5th. | Totals. |
|---|---|---|---|---|---|
| Winifred | — | 672 | Sunshine | 23 | 1150 |
| Adelaide | — | 646 | Electrician | 23 | 1291 |
| Nettie Vance | — | 757 | H. C. | — | 562 |
| F. W. P. H. | 26 | 1340 | Reldas | 25 | 1353 |
| Amos | — | 596 | Rover | — | 776 |
| Reyot | — | 369 | Qu'appelle | 21 | 1094 |
| Ellice | 24 | 1369 | Beryl | — | 188 |
| A. S. K. | — | 275 | Lightowlers | 22 | 1392 |
| Neptune | 22 | 1276 | Hercules | — | 12 |
| Ecila | 26 | 1427 | Jenny Wren | 21 | 961 |
| Tinie | — | 17 | Night Watch | — | 346 |
| Amicus | — | 946 | Oakenrod | — | 1122 |
| Juvenis | — | 539 | Embryo | — | 385 |
| Jumps | 25 | 1420 | Gypsye | — | 967 |
| Sister | — | 438 | Leo | — | 676 |
| M. W. | 26 | 1364 | Pearl | — | 299 |
| Esperance | 26 | 1363 | Africando | — | 171 |

### Notice to Correspondents.

**N.B.—All letters referring to this page which do not arrive at 139 and 140, Salisbury-court, London, E.C., by the *first post on Thursdays*, and are not addressed PRIZE EDITOR, will in future be disqualified and disregarded.**

